# High‐Performance and Reliable White Organic Light‐Emitting Fibers for Truly Wearable Textile Displays

**DOI:** 10.1002/advs.202104855

**Published:** 2022-01-24

**Authors:** Yong Ha Hwang, Byeongju Noh, Junwoo Lee, Ho Seung Lee, Yongjin Park, Kyung Cheol Choi

**Affiliations:** ^1^ School of Electrical Engineering Korea Advanced Institute of Science and Technology Daejeon 34141 Republic of Korea

**Keywords:** fiber OLED, fiber WOLED, textile displays, wearable displays

## Abstract

Light‐emitting fibers have been intensively developed for the realization of textile displays and various lighting applications, as promising free‐form electronics with outstanding interconnectivity. These advances in the fiber displays have been made possible by the successful implementation of the core technologies of conventional displays, including high optoelectronic performance and essential elements, in the fiber form‐factor. However, although white organic light‐emitting diodes (WOLEDs), as a fundamental core technology of displays, are essential for realizing full‐color displays and solid‐state lighting, fiber‐based WOLEDs are still challenging due to structural issues and the lack of approaches to implementing WOLEDs on fiber. Herein, the first fiber WOLED is reported, exhibiting high optoelectronic performance and a reliable color index, comparable to those of conventional planar WOLEDs. As key features, it is found that WOLEDs can be successfully introduced on a cylindrical fiber using a dip‐coatable single white‐emission layer based on simulation and optimization of the white spectra. Furthermore, to ensure durability from usage, the fiber WOLED is encapsulated by an Al_2_O_3_/elastomer bilayer, showing stable operation under repetitive bending and pressure, and in water. This pioneering work is believed to provide building blocks for realizing complete textile display technologies by complementing the lack of the core technology.

## Graphical abstract

Although the core technologies of organic light‐emitting devices (OLEDs) have been implemented in the form of fiber, the fiber WOLED, a core technology to realize full‐color displays and various applications, is still undeveloped. A fiber WOLED not only was reported for the first time but also exhibited a high level of optoelectronic performance, reliable color index, deformability, and durability.

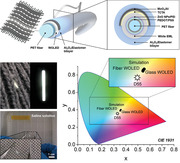

## 1 Introduction

The continued interest of consumers in wearable devices that enhance the quality of life (QoL) has led to rapid advances in the form‐factors of devices, such as flexible,^[^ ^1^, ^2^, ^3^ ^]^ foldable,^[^ ^4^ ^]^ and stretchable electronics,^[^ ^5^, ^6^ ^]^ which can increase the information‐accessibility and convenience while maintaining the existing functions and performance. This immense interest has triggered much research on electronic textiles (e‐textiles),^[^ ^7^, ^8^, ^9^ ^]^ one of the most promising free‐form factors with transcendent interconnectivity between humans and devices, which are intended to transcend the physical and conceptual limitations of conventional wearable devices, in that the technologies can be truly worn like daily clothes. In particular, for various applications, including energy devices,^[^ ^10^, ^11^ ^]^ sensors,^[^ ^12^, ^13^ ^]^ switches,^[^ ^14^, ^15^ ^]^ circuits,^[^ ^16^ ^]^ and light‐emitting devices,^[^ ^17^ ^]^ fiber‐based electronics (fibertronics), have attracted considerable attention as an advanced form‐factor due to their distinct advantages resulting from their cylindrical geometry, which is different from other free‐form factors.^[^ ^18^ ^]^ However, the cylindrical form of fibers has both advantages and disadvantages. Unlike conventional 2D planar devices, there are not only functional advantages, including lightweight and breathability, but also differentiated deformability such that they can withstand various user‐induced curvatures because of their flexibility in all directions. However, this results in difficulty in implementing devices on cylindrical fiber.^[^ ^19^ ^]^ Thus, it is very important to simultaneously realize these distinguishing advantages of fibers and the successful implementation of the core technologies, including optoelectronic performance and essential elements, comparable to those of conventional planar devices.

This importance is also a major challenge without exception in the field of fiber‐based displays, which can provide visualized information with superior accessibility and convenience via the integration of daily clothing and devices. In particular, in terms of the technical maturity that can fully realize high‐resolution and full‐color displays, it has been important to realize light‐emitting fibers with the core technologies, including optoelectronic performance, fundamental functions, and essential elements, comparable to those of conventional planar organic light‐emitting diodes (OLEDs), which are the commercially proven industry‐leading devices that can surpass other light‐emitting devices, such as electrochemical cells (LECs),^[^ ^20^, ^21^ ^]^ alternating‐current electroluminescence (ACEL) devices,^[^ ^22^, ^23^ ^]^ inorganic light‐emitting diodes (LEDs),^[^ ^24^, ^25^ ^]^ and quantum dots (QDs).^[^ ^26^ ^]^ In this regard, there have been considerable efforts and notable breakthroughs to implement the core technologies and achieve good performance, including sufficient brightness with low driving voltages, high current efficiency (CE), and an addressable scheme, comparable to those of conventional planar OLEDs.^[^ ^27^, ^28^, ^29^, ^30^, ^31^, ^32^ ^]^ In particular, among previous works, several fiber OLEDs using the dip‐coating method, which is capable of uniformly depositing films on cylindrical structures, have been reported.^[^ ^29^, ^30^, ^31^, ^32^ ^]^ In these previous works using the dip‐coating method, including our previous works and other works, essential display technologies, including fluorescent OLEDs, phosphorescent OLEDs, a light‐extracting technique, and a communication system were implemented on fibers.^[^ ^26^, ^29^, ^30^, ^31^, ^32^ ^]^ To realize these notable breakthroughs based on the dip‐coating method, the researchers fully focused on each device structure design, and its purpose, ensuring suitability for each light‐emitting device. However, although white organic light‐emitting diodes (WOLEDs) are an essential core technology in displays for realizing full‐color displays and solid‐state lighting applications, WOLEDs on fibers are still undeveloped because there were no suitable structural studies or approaches for implementing WOLEDs on fiber, and the dip‐coating method does not unconditionally ensure successful implementation and performance comparable to the outcomes of planar devices. In particular, given that WOLEDs feature complex structures and sensitive properties with regard to white emissions, suitable structure designs and approaches for implementing fiber WOLEDs comparable to those of planar devices are still challenging. In addition, durability against external stimuli, including sweat, pressure, and the risk of contact, which can be encountered with the daily use of clothing, was still not considered.

Conventional WOLEDs, which are based on tandem structures that have two or three times as many emission layers as those in normal OLEDs,^[^ ^29^ ^]^ are difficult to realize on cylindrical fibers. The reason is that multiple layers, which are thermally deposited on the cylindrical fiber, induce many uneven thin layers at the edges of cylindrical fibers. Importantly, it is known that the uneven thin layers on the edges cause instability of devices.^[^ ^26^ ^]^ In particular, this fact suggests that a tandem structure with an ultra‐thin charge generation layer (CGL) may be even more fatal. In the case of WOLEDs with a single emission layer, although they feature a small number of layers in comparison to tandem structures, the vacuum thermal deposition of multiple materials including hosts and dopants at the same time leads to significant complexity and cost‐effectiveness problems in terms of the manufacturing process, equipment, and materials.^[^ ^33^ ^]^ These problems may be resolved through a solution process.^[^ ^33^ ^]^ However, as with the vacuum thermal evaporation method, it is difficult to obtain a reliable color index.^[^ ^34^, ^35^ ^]^ Hence, an optimized strategy is required to realize WOLEDs that have reliability and can be stably stacked on a cylindrical fiber substrate.

As described above, in terms of the usage of fiber OLEDs, ensuring their durability is essential because they are exposed to external stimuli in daily life, such as sweat, pressure, and so on. In this regard, a novel approach was suggested in which a woven scheme consisting of process‐friendly rectangular stripe‐typed fiber OLEDs can be operated in water by introducing a polyurethane (PU)‐based passivation system using a stamp‐assisted printing method.^[^ ^36^ ^]^ As an advanced form‐factor, cylindrical fiber OLEDs require a method to ensure durability that would allow stable operation under exposure to external stimuli as well as successful implementation on cylindrical fibers.

In this paper, we present the first fiber‐based white organic light‐emitting diodes (fiber WOLEDs) fabricated with a dip‐coatable single white emission layer (single white EML) and encapsulation by an Al_2_O_3_/elastomer bilayer, resulting in high performance and reliable color index comparable to those of the conventional planar WOLED and durability against external stimuli, including sweat, pressure, and risk of contact, induced by daily use of clothing. As a result of careful consideration of the device structure, simulation, and optimization of the white emission spectra for the implementation of WOLEDs on cylindrical fibers with a diameter of 250 µm, the proposed fiber WOLEDs exhibited a high brightness (≈738 cd m^–2^) and a CE value (≈10.8 cd A^–1^) at low driving voltages (<6 V), a color‐shift with little change, and a high color rendering index (CRI) value of ≈80, which are comparable to those of conventional planar WOLEDs. In addition to high performance and a reliable color index, the geometric advantages of the cylindrical fiber were confirmed by 1000 cyclic bending tests under 1.5% tensile strain. Furthermore, the fiber WOLED, which was encapsulated by an Al_2_O_3_/elastomer bilayer, showed a stable optical property, which was confirmed by measurement of the optical property and optical simulation. Moreover, it showed durability such that it could be stably operated in a saline solution similar to sweat and survive under repeated application of pressure conditions.

## 2 Discussion and Result

**Figure 1:**
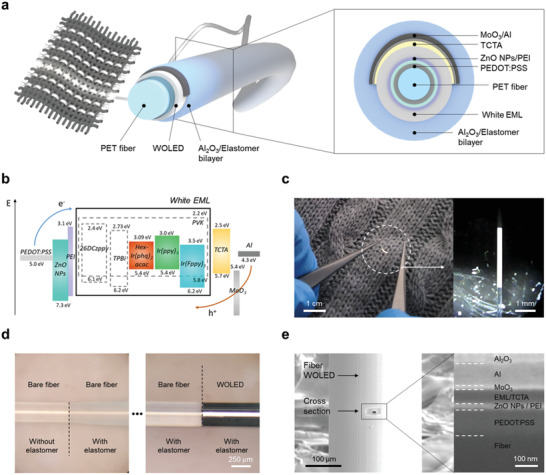
a) Schematic illustration of a fiber WOLED and its cross‐section. b) Energy‐level diagram of the fiber WOLED. c) Photograph of the proposed fiber WOLED (left) and microscopic images (right) of it in operation at 4.0 V bias. d) Photographs of the fiber WOLED with elastomer encapsulation. e) Magnified view of the fiber WOLED obtained by scanning electron microscopy (SEM) (left) and a cross‐sectional SEM image of the WOLED on the fiber showing multi‐stacked layers (right).

**Figure** **1**a presents a schematic illustration of a fiber‐based white organic light‐emitting diode (fiber WOLED) and a cross‐sectional view, showing the configuration of the WOLED on a transparent PET fiber with a diameter of 250 µm. The layers including a transparent and flexible poly(3,4‐ethylenedioxythiophene):‐polystyrenesulfonate (PEDOT:PSS) cathode and a zinc oxide (ZnO)/polyethylenimine (PEI) electron injection layer (EIL)^[^ ^37^ ^]^ were formed by a dip‐coating method, which can uniformly deposit these films on cylindrical structures, as previously reported.^[^ ^30^ ^]^ Next, a single white emission layer (single white EML) was evenly introduced upon the EIL by the dip‐coating method. The other layers including 4,4′,4″‐tris(carbazol‐9‐yl)‐triphenylamine (TCTA), which simultaneously act as an electron blocking layer (EBL) and a hole transport layer (HTL), a molybdenum oxide (MoO_3_) hole injection layer (HIL), and an aluminum (Al) anode, were thermally deposited in one direction of the fiber (Figure S1, Supporting Information). Subsequently, as needed, the fiber WOLED was encapsulated by Al_2_O_3_ by atomic layer deposition (ALD), a chemical vapor deposition (CVD) method, using vapor‐type precursors, thereby enabling it to be deposited on the entire surface of the fiber. As seen in Figure 1b, the single white EML consisted of red, green, and blue (RGB) components to achieve a high color gamut. In detail, the single white EML was composed of host materials, namely, poly(*N*‐vinyl carbazole) (PVK) polymer, 2,6‐bis(3‐(9‐carbazol‐9‐yl)phenyl)pyridine (26DCzppy), and 1,3,5‐tris(*N*‐phenylbenzimidazol‐2‐yl)benzene (TPBi), and dopant materials, namely, bis[2‐(4‐*n‐*hexylphenyl)quinoline](acetylacetonate)iridium(III) (Hex‐Ir(phq)_2_acac), tris(2‐phenyl pyridine)‐iridium(III) (Ir(ppy)_3_), and tris[2‐(2,4‐difluorophenyl)pyridine]iridium(III) (Ir(Fppy)_3_) (Figure S2, Supporting Information). The PVK host acts as the bind host and provides sufficient surface tension for application of the dip‐coating method.^[^ ^26^, ^32^ ^]^ The other host material was carefully selected to achieve charge balance. To fabricate the dip‐coatable single white EML, each RGB solution was mixed in an appropriate and carefully selected volume ratio, which will be discussed in detail later. Figure 1c presents the fiber WOLEDs in operation at a 4 V bias, including the white emission of the fiber WOLED and the transparent PET fiber. Figure 1d shows the fiber WOLED encapsulated by an elastomer (≈17 µm) fabricated by the dip‐coating method. Figure 1e shows a magnified image of the fiber WOLED and its cross‐sectional view from the cathode to the Al_2_O_3_ layer obtained by using a focused ion beam (FIB) system with scanning electron microscopy (SEM), confirming the thicknesses of the uniform layers: the PEDOT:PSS (≈120 nm), the ZnO/PEI bilayer (≈15 nm), the EML (≈20 nm), TCTA (≈40 nm), MoO_3_ (≈10 nm), Al (≈100 nm), and Al_2_O_3_ (≈50 nm). As shown in Figure 1a,b, as a key feature to solve the aforementioned challenges that have so far precluded the implementation of WOLEDs on cylindrical fiber, the proposed fiber WOLED features the dip‐coated single white EML. The dip‐coated single white EML is the most suitable design for realizing a WOLED on cylindrical fiber, allowing the use of fewer layers of the WOLED in comparison to tandem structures and avoiding the aforementioned instability issue resulting from the uneven film of the edge, simultaneously. However, to achieve a reliable color index, it was also necessary to optimize the white color spectra in addition to the implementation of the WOLED.

**Figure 2:**
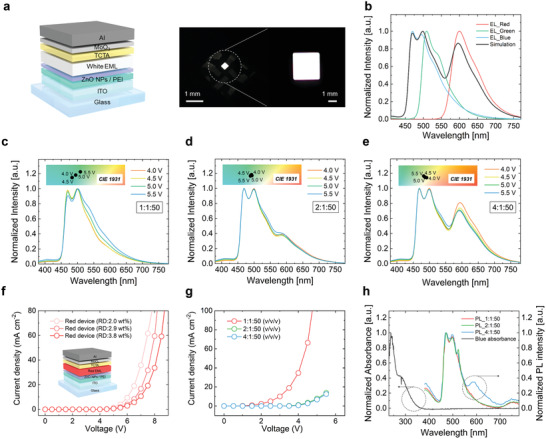
a) Schematic illustration of ITO‐coated glass‐based WOLED (left) and a photograph and microscopic image of the glass‐based WOLED (right). b) Electroluminescence (EL) spectra of red, green, and blue devices and the simulation result c–e) EL spectra according to the red, green, and blue solution mixture ratio. RGB volume ratios: 1:1:50 (c), 2:1:50 (d), and 4:1:50 (e) (inset: color coodinates in Commission International d'Echairage (CIE) 1931 color coodinates). f) Current density‐voltage (*J*–*V*) curves of red devices according to the doping ratio (inset: illustration of a red OLED). g) *J*–*V* curves of (c), (d), and (e). h) Normalized absorbance of the blue dopant and photoluminescence (PL) intensity of (c), (d), and (e), resulting from an exposition of light (238 nm) at the blue absorbance wavelength.

Prior to the realization of the WOLED on the fiber, the white emission was optimized in an indium tin oxide (ITO)‐coated glass‐based WOLED (glass WOLED), based on the simulation and experimantal optimization of the white color spectra. **Figure** **2**a presents a photographic image of the glass WOLED and its illustration, showing the device structure of the glass WOLED consisting of the same layers as the fiber WOLED except for the PEDOT:PSS cathode, which was replaced by an ITO cathode. To establish the components and characteristics of the white EML for white emission with a high and reliable color index, the white color spectra was first simulated. Under the simple assumption that there is no cavity effect and no formation of the exciplex in the WOLED, white electroluminescence (EL) intensity can be expressed as the linear combination of each RGB EL intensity that acts as a basis (Figure S3, Supporting Information). Thus, the white color that is closest to daylight white (D55 in Commission International d'Echairage (CIE) 1931), based on each RGB EL intensity, was calculated (Figure 2b), resulting in the fact that a spectral similarity index (SSI) value of the fiber WOLED is ≈71.

Based on the simulation result, the white color spectra was experimentally optimized to ensure a reliable white emission while achieving a spectra similar to the simulated white color spectra. The white emission of the WOLED based on the single white EML consists of competition between two emission mechanisms, namely, energy transfer (ET) from the blue dopant to the red dopant and charge trapping (CT) to the dopants.^[^ ^38^, ^39^ ^]^ Thus, with these characteristics in mind, a white solution for the single white EML was prepared with a large proportion of a blue solution when RGB solutions were mixed. For example, in the case of an unsuitable volume ratio in which red, green, and blue solutions had an equal volume ratio (1:1:1), it could be seen that the ET was dominant in the direction of low energy; therefore, blue emission did not occur, but yellowish emission occurred (Figure S4, Supporting Information). In addition to the consideration of dominant blue, to attain reliable white emission, it is also important to make one of two emission mechanisms dominant or to suppress the CT because it is known that these issues affect the color shift according to the driving voltage.^[^ ^40^, ^41^ ^]^ To this end, it was necessary to understand that the emission mechanism in WOLEDs with single EMLs is mostly determined by red dopants with low energy states, which act as electron traps, resulting in the CT.^[^ ^38^ ^]^ In our case as well, the characteristic of the red dopant was verified by the fact that the red device using the red dopant showed changes in the current density‐voltage (J‐V) curve depending on the applied voltages, as shown in Figure 2f (Figure S5, Supporting Information). Therefore, it was reasonably expected that our WOLED using the red dopant would have the emission of the CT induced by the red dopant. Both the ET and the CT in WOLEDs with single EMLs are ideally proportional to a certain level of the doping ratio of the red dopant, resulting from variations in the distance between dopants and the number of traps (dopants), respectively.^[^ ^34^ ^.^ ^42^ ^]^ However, because each proportion depending on the doping ratio is different, the dominant point of each mechanism can be different.^[^ ^43^, ^44^ ^]^ In our WOLED, by finding the dominant point of the ET, it was possible to achieve a reliable color index as well as a high color index that is closest to daylight. Figure 2c,d,e show color spectra data according to the mixing ratios of the RGB solutions and applied driving voltages in each WOLEDs. Also, it was found that the main red peak (≈600 nm) appeared when the ratio of the red solution was increased. In particular, it was worth noting that the color shift depending on the applied voltages significantly decreased with other volume ratios (2:1:50 and 4:1:50), compared to the volume ratio (1:1:50). These results can be explained by the aforementioned solution in which the ET is dominant. As shown in Figure 2g, the *J*–*V* curve changed when the volume ratio of the red solution was increased from 1 to 2, but there was no change in the *J*–*V* curve when the volume ratio of the red solution was increased from 2 to 4. This means that the CT is dominant with the volume ratio (1:1:50), but the ET is dominant with the other volume ratios (2:1:50 and 4:1:50). To further verify the prediction, photoluminescence (PL) analysis was also performed (Figure 2h). It is generally known that the PL consists of only the ET without the CT. Thus, if the CT is dominant, it can be expected that there would be no change between the PL peak (≈600 nm) of red with the volume ratio (1:1:50) and that with the volume ratio (2:1:50), while the EL peaks (≈600 nm) of red consisting of both the CT and the ET increased as seen in Figure 2c,d. Unlike the comparison of the volume ratio (1:1:50) and the volume ratio (2:1:50), in the comparison of the volume ratio (2:1:50) and the volume ratio (4:1:50), the PL peak (≈ 600 nm) of red significantly increased, meaning that the ET significantly increases. In the additional analysis, it could be clearly confirmed that the ET is dominant with the volume ratio (2:1:50) and the volume ratio (4:1:50). In the two cases of the dominant ET, the white color spectra with the volume ratio (4:1:50) were similar to the simulated white color spectra. Therefore, with the volume ratio (4:1:50), a white color spectra with a high and reliable color index was realized.

**Figure 3:**
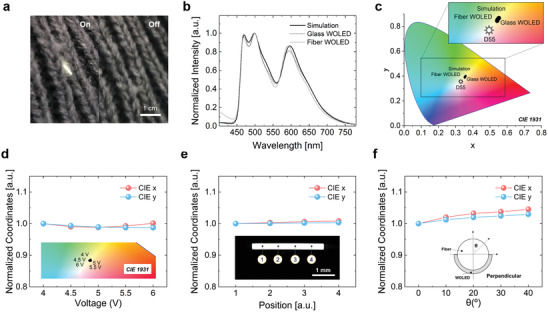
a) Demonstration of the fiber WOLED, which was integrated into daily clothing and operated at 4.0 V. b) Comparison of EL spectra of the glass WOLED, the fiber WOLED, and the simulation result. c) CIE (*x*,*y*) coordinates of the glass WOLED, the fiber WOLED, and the simulation result (Sun symbol means D55, which is daylight white in CIE 1931). d) Normalized CIE (*x*,*y*) coordinates of the fiber WOLED at various voltages (inset: CIE 1931 color coordinate). e) Normalized CIE (*x*,*y*) coordinates of the fiber WOLED at various positions (inset: microscopic image of the fiber WOLED). f) Normalized CIE (*x*,*y*) coordinates of the fiber WOLED at various perpendicular angles (inset: perpendicular view of the fiber WOLED).

As a result of careful consideration of the device configuration that would be suitable for cylindrical fiber, such as the dip‐coatable bind material, as well as considerations for charge balance, and the configuration of the white EML, the fiber WOLED successfully exhibited white emission at a low turn‐on voltage (4 V bias), which could be integrated into daily clothing by the hand‐weaving method (**Figure** **3**a). Figure 3b presents the simulation result, the EL spectrum of the glass WOLED, and the measured EL spectrum of the fiber WOLED. Consistent results were achieved with no significant difference with the main peak wavelengths, including red (600 nm), green (509 nm), and blue emission (470 nm). Figure 3c also presents the color index without significant changes between the CIE color coordinates of each result. Based on the white color spectra of the fiber WOLED, the high color rendering index (CRI) value of ≈80 was calculated (Table S2, Supporting Information). As seen in Figure 3d,e,f, due to the simulation and the experimental optimization of the white color index, the fiber WOLED exhibited reliable emission in color coordinates according to the driving voltages, positons, and perpendicular angles. The fiber WOLED exhibited white emission with little change (maximum Δ*x* = 0.00413 and Δ*y* = 0.00497, and the maximum rate of change Δ*x* = 1.1% and Δy = 1.2%) from 4 to 6 V, as shown in the inset of Figure 3d. As with the results according to the driving voltages, at various positions of the fiber WOLED, reliable white emission was confirmed, as seen in Figure 3e (maximum Δ*x* = 0.00257 and Δ*y* = 0.00144, and the maximum rate of change Δ*x* = 0.8% and Δ*y* = 0.3%). Also, white emission with no significant changes according to the perpendicular angles is shown in Figure 3f (maximum Δ*x* = 0.01494 and Δ*y* = 0.01200, and maximum rate of change Δ*x* = 4.5% and Δ*y* = 2.9%).

**Figure 4:**
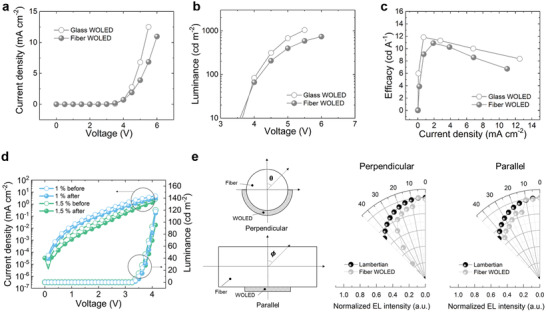
a‐c) Current density‐voltage‐luminance (*J*–*V*–*L*) curves of the glass WOLED and the fiber WOLED d) *J*–*V*–*L* curves of the fiber WOLED before and after repetitive 1000‐cycle bending tests. e) Measured EL intensity in the perpendicular (*θ*) and parallel (*ф*) directions.

With reliable white emission, the fiber WOLED showed high optoelectronic performance including brightness with low driving voltages and current efficiency (CE), comparable to those of the conventional planar WOLED based on the ITO electrode. **Figure** **4**a–c presents the current density‐voltage‐luminance (*J*–*V*–*L*) curves of the fiber WOLED, which are comparable to that of the conventional WOLED, including a high brightness value of 738 cd m^–2^, the CE value of 10.8 cd A^–1^, and a low operation voltage of <6 V (Table S1, Supporting Information). However, with regard to brightness, there is a difference between the glass WOLED and the fiber WOLED stemming from the different refractive indices, the different conductivity levels, the transmittance of the substrates, in this case glass and PET, and the electrodes, in this case ITO and PEDOT:PSS (Figure S7, Figure S8, and Figure S12, Supporting Information). Having achieved optoelectronic performance comparable to that of the conventional WOLED as well as a reliable color index, to verify the advantages of the fiber form‐factor and evaluate the deformability of the fiber WOLED, a repetitive 1000‐cycle bending test was performed. Under the pure bending assumption, the tensile strain that is applied to a device is determined by the bending radius (*R*) and thickness of the substrate (*t)*.^[^ ^30^ ^]^ Thus, it can be seen that the fiber could withstand up to 1.5% tensile strain after the 1000‐cycle bending test (Figure 4d and Figure S6, Supporting Information), meaning that the fiber WOLED can be woven into daily clothing based on its flexibility and light weight. Figure 4e shows the geometry on the emission profile and EL intensity according to the emission angle of the WOLED in two directions (*θ*, *ϕ*), namely, perpendicular and parallel, respectively. In the *ϕ* direction, the EL intensity of the fiber WOLED showed an angular spectrum similar to a Lambertian emission. Meanwhile, the angular spectrum of the perpendicular direction differed greatly depending on the angle. In addition to the reinforced emission induced by the circular shape of the fiber, this result can be attributed to the fact that the thin and non‐uniform lateral surface of the thermally deposited layers induced an imbalance of the electron‐hole charge, resulting in reduced light emission as opposed to when a uniform thermal deposited film is used (Figure S7 and Figure S8, Supporting Information).^[^ ^30^ ^]^

**Figure 5:**
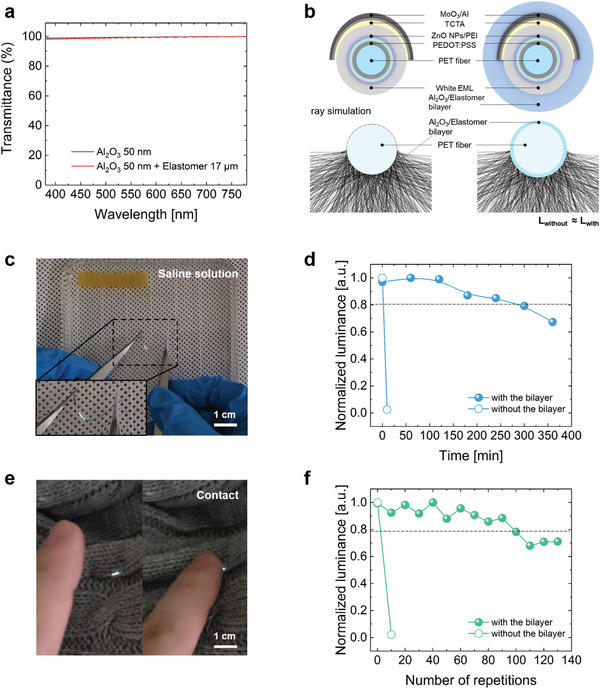
a) Transmittance of the Al_2_O_3_ single layer and the Al_2_O_3_/elastomer bilayer. (Baseline: a glass substrate). b) Simulated outcoupling efficiency of the fiber WOLED with or without the Al_2_O_3_/elastomer bilayer obtained by using a ray‐tracing simulation tool (LightTools) (*L*_without_ or *L*_with_: number of rays in the case of the fiber WOLED without or with the bilayer). c) Photograph of the Al_2_O_3_/elastomer bilayer‐encapsulated fiber WOLED in a saline solution (yellowish paper: a litmus paper for checking pH). d) Changes in luminance over time in a saline solution. e) Photograph of the Al_2_O_3_/elastomer bilayer‐encapsulated the fiber contacted by a finger. f) Luminance values according to the number of times it was pressed.

As well as high optoelectronic performance and flexibility which are important to achieve visualization‐interactivity between humans and machines, durability to make this interactivity sustainable is essential. Therefore, to obtain validity beyond limited usability, the fiber WOLED should be resistant to oxygen sweat, and pressure, and should show biocompatibility when exposed to these external stimuli encountered in daily life. Thus, to further advance the potential of the fiber WOLED by achieving durability, the fiber WOLED with the Al_2_O_3_ layer was dip‐coated in an elastomer solution (Ecoflex) which is known for its hydrophobic property and biocompatibility.^[^ ^45^ ^]^ Meanwhile, prior to verifying its durability as the outmost layer of the fiber WOLED, it was necessary to confirm that the elastomer layer rarely degrades the optical performance. To confirm this fact, the transmittance was measured, and the outcoupling efficiency was simulated. As shown in **Figure** **5**a, the overall transmittance of the bilayer was about 98.5% from 380  to 780 nm. In addition, to predict the quantitative light loss, the optical simulation based on the ray‐tracing was executed in the two cases; without the bilayer and with the bilayer that set to the transmittance of 98.5% (Figure 5b and Figure S7, Supporting Information). As a result of the simulation, a similar number of rays was confirmed regardless of the bilayer, meaning that the outcoupling efficiency can be maintained. Furthermore, the measured efficiencies, which were confirmed to be similar, indicate that the optical property is generally maintained (Figure S9, Supporting Information).

In addition to the fact that the optical property is rarely impaired, durability against the stimulation that can be encountered in daily use was verified. To estimate sweat‐resistance, the fiber WOLED was immersed in a saline solution with a salty and slightly acidic pH similar to human sweat. By checking for dark spots with a microscope, it was confirmed that the encapsulated fiber WOLED could be reliably operated without dark spots for about 300 min due to the hydrophobicity of the elastomer (Figure 5d). This finding suggests that the fiber WOLED can be operated reliably in various environmental conditions that can arise during routine use, such as sweat and raindrops, among others (Movie S1, Supporting Information) considering the fact that the fiber WOLED without encapsulation did not survive in a saline solution and air, unlike the encapsulated fiber WOLED, showing an LT_50_ value (initial luminance: 50 cd m^−^^2^) of approximately two hours (Figure S13, Supporting Information). In addition to sweat‐resistance, to evaluate the fiber WOLED's durability against the physical stress that can occur in daily use, a repetitive pressure test was performed (Figures 5e,f). As shown in Figure 5e, it can be seen that fiber WOLED operated stably even after the application of 100 cycles of a load condition of ≈1 N, which is a stronger intensity than when touching a touch screen.^[^ ^46^ ^]^

Although the proposed fiber WOLED exhibited durability in comparison to previous works, advanced durability is required to realize robust platforms, in that durability decreases rapidly under harsh conditions (Figure S10, Supporting Information). Obviously, it is easy to predict that as the thickness of the elastomer increases, durability is also enhanced. However, increasing thickness may impair the optical property due to other issues such as uneven transmittance (Figure S11, Supporting Information). To address this trade‐off, a thin polymer with excellent robustness, such as a polymer applied by the initiated chemical vapor deposition (iCVD) process, could be a suitable alternative.^[^ ^47^ ^]^ Also, the WOLED was successfully implemented on the fiber and exhibited great potential for practical applications in daily clothes, but the weaving direction may be limited due to the emission property, i.e., light being emitted in only one direction. It is possible to weave in a limited direction so that light can be emitted outward from the clothes, but there is a practical suggestion that can fundamentally resolve this limitation. The potential option, as an alternative to a thick anode, is to use transparent electrodes using the dip‐coating method, such as conductive polymers and transparent silver wires, that provide the emission property of illumination in all directions.^[^ ^48^ ^]^ Furthermore, the potential option can solve the problem of reduced emitted light induced by the charge imbalance from the thin side layers of the fiber. This potential option may provide future opportunities that can expand the limited applications of the fiber WOLEDs.

## 3 Conclusion

In summary, we reported the first fiber WOLED enabled by a single emission layer fabricated by the dip‐coating method to overcome limitations including geometry and device configuration. The proposed fiber WOLED exhibited not only high optoelectronic performance and reliable color index comparable to those of the conventional WOLED, but also durability, including sweat‐resistance, pressure‐resistance, and biocompatibility. The WOLED with a dip‐coated single emission layer consisting of RGB components was implemented on a cylindrical fiber with a diameter of 250 µm. As a result of the simulation and optimization of the white color spectra, the fiber WOLED showed a white color index including the CRI value of ≈80 and a color index with little change in the color coordinates (Δ*x* = 0.00413 and Δ*y* = 0.00497, from 4  to 6 V). The fiber WOLED showed a brightness value of ≈738 cd m^–2^, a current efficiency value of ≈10.8 cd A^–1^, and a low operation voltage of <6 V. Additionally, after a flexibility test of the fiber WOLED with 1000 cycles under a tensile strain of 1.5%, the fiber WOLED could be stably operated. Furthermore, to attain durability including sweat‐resistance and contact‐protection from external stimuli, the fiber WOLED, was encapsulated in Al_2_O_3_/elastomer. The device stably functioned in a saline solution for 300 min without dark spots and reliably operated under 100 cycles of application of load conditions (≈1 N) with little change.

Given that the first fiber WOLED exhibited a comparable level of high optoelectronic performance, reliable color index, and deformability offered by the nature of the fiber, and durability for daily use, we believe that this pioneering work, as a complement to conventional core technology in the field of fiber‐based displays, provides building blocks for future research to realize truly wearable displays (i.e., wearing displays) in the form of a complete platform.

## 4 Experimental Section

### Preparation of Solutions

To prepare the white EML solution, red, green, and blue EML, which consisted of poly(*N*‐vinylcarbazole) (PVK), 6‐bis(3‐(9H‐carbazol‐9‐yl)phenyl)pyridine (26DCzppy), 1,3,5‐tris(*N*‐phenylbenzimidazol‐2‐yl)benzene (TPBi), Bis[2‐(4‐n hexylphenyl)quinoline](acetylacetonate)iridium(III) (Hex‐Ir(phq)_2_(acac)), tris(2‐phenyl pyridine)‐iridium(III) (Ir(ppy)_3_), and Tris(2‐(4,6 ‐difuorophenyl)pyridine)iridium(III) (Ir(Fppy)_3_) were prepared by the methods used in the previous work.^[^ ^32^ ^]^ Then, the red, green, and blue solutions were mixed in vials (in a 1:0.25:12.5 volume ratio). A PEDOT:PSS solution, a ZnO NPs solution, and a PEI solution were prepared by the methods used in the previous work.^[^ ^30^ ^]^ The PEDOT:PSS solution was composed of PH1000 solution (Clevios), dimethyl sulfoxide (DMSO), and Zonly FS‐300 (94.5:5:0.5 weight ratio) (Figure S12, Supporting Information). To synthesize ZnO NPs, 1.51 g of KOH was dissolved in 60 mL of methanol (MeOH). Next, a zinc acetate dehydrate (Zn(Ac)_2_·2H_2_O) solution was prepared by dissolving 3 g of Zn(Ac)_2_·2H_2_O in 120 mL of MeOH. After these solutes were completely dissolved, the KOH solution was added dropwise to the Zn(Ac)_2_·2H_2_O solution, which was placed in a round‐bottom flask. The mixture in the flask was stirred for 100 min, and then it was centrifuged at 4000 rpm for 20 min. Finally, the resulting ZnO NPs were re‐dispersed in 1‐butanol at 2 wt%. Polyethylenimine (PEI) was diluted in 99.6 wt% 2‐methoxy ethanol. The saline solution was composed of 9 g of sodium chloride (NaCl) in 1000 mL of distilled water (JW‐LifeScience Inc.).

### Fabrication of the Fiber WOLED

PET fibers with a diameter of 250 µm were prepared by a cleaning process. After cleaning with isopropyl alcohol (IPA) and deionized water (DI water) by sonication, a PET fiber was dip‐coated in the PEDOT:PSS solution, the ZnO NPs solution, the PEI solution, and the single white emission solution in sequence. The dip‐coating speeds for each solution were 0.7 mm s^–1^, 2 mm s^–1^, 10 mm s^–1^, and 50 mm s^–1^. All of the solution processes were implemented under an atmospheric pressure and nitrogen environment. Also, the annealing time for each stage was 30 min, 30 min, 10 min, and 20 min at 100 ℃. Then, 4,4′,4′′‐tris(*N*‐carbazolyl)triphenylamine (TCTA), molybdenum oxide (MoO_3_), and aluminum (Al) were thermally deposited by evaporation at 3 × 10^–6^ torr.

### Encapsulation of the Fiber WOLED

For the encapsulation process, an Al_2_O_3_ layer (50 nm) was deposited by an atomic layer deposition (ALD) machine (LUCID D100, NCD Inc), which alternately dispensed trimethylaluminum (TMA) and H_2_O at 70 ℃ (pulse time: 0.2 s and purge time: 10 s). In the ALD chamber, to ensure coverage of the entire surface, the fiber WOLED was located between the floor and the ceiling of the chamber. Next, Ecoflex, which consists of a prepolymer A and prepolymer B (1:1 volume ratio) layer, was deposited by dip‐coating at 0.1 mm s^–1^.

### Simulations

A ray‐tracing simulation was performed to obtain the outcoupling efficiency using the commercial software LightTools (Synopsis Inc.) (Figure S7, Supporting Information). LightTools was also used to calculate the CRI value. The simulated white wavelength was obtained by custom‐made MATLAB codes under the aforementioned simple assumptions (Figure S3, Supporting Information). A value of the SSI was obtained by the Academy Spectral Similarity Index Calculator (Academy of Motion Picture Arts and Sciences (AMPAS.)).

### Device Characterization

A cross‐section scanning electron microscopy (SEM) image of the multilayer was acquired using a focused ion beam (FIB) system (Quanta 3D FEG, USA) and an FE‐SEM (S‐4800, Hitachi Inc.), located in the KAIST Analysis Center for Research Advancement (KARA). The PL spectra were measured by fluorescence spectrometry (FS‐2, Scinco Inc.). A UV–vis spectrophotometer (UV‐2550, Shimadzu Inc.) was used to measure the absorbance of the blue dopant and the transmittance of the thin film bilayer and the PEDOT:PSS electrode. A source meter (2400 series, Keithley Inc.) and a spectroscopic radiometer (CS2000, Konica Minolta Inc.) equipped with a close‐up lens (CS‐A35) were used to measure the current density‐voltage‐luminance (J‐V‐L). Also, a Ф 0.1 mm diameter of the measurement spot provided by the close‐up lens and a 0.1° measurement angle were used. The light‐emitting area was computed using the following equation: *π* × *r* × *L*, where r and *L* represent the radius of the cylindrical fiber substrate and the length of the fiber WOLED, respectively. The cyclic bending test was performed using a bending test machine (Sciencetown Inc.). Weights of 100 g and 500 g were used to apply repetitive pressure conditions. The thickness of the elastomer on the fiber was measured by an alpha‐step machine (KLA‐Tencor ASIQ).

### Statistics

The output performance (luminance, current, and voltage) were all adopted from the measured original data using the aforementioned measurement machines. Based on the calculated light‐emitting area, which was mentioned above, the current density was computed. The output data of the fiber WOLED were chosen as the data closest to the average among the 10 sets of data. All of the normalized data were normalized based on the highest value of each data. All of the curves were programmed by the software (Origin).

## Conflict of Interest

The authors declare no conflict of interest.

## Supporting information

Supporting Information

Supplemental Movie 1

## Data Availability

The data that support the findings of this study are available from the corresponding author upon reasonable request.
