# Supplementary material for: High‐Performance and Reliable White Organic Light‐Emitting Fibers for Truly Wearable Textile Displays
Source: Adv Sci (Weinh). 2022 Jan 24;9(11):2104855. doi: 10.1002/advs.202104855 (PMC9008425; doi:10.1002/advs.202104855)
Supplement: Supplementary file 1 — Supporting Information [file ADVS-9-2104855-s002.pdf]

ADVANCED SCIENCE
Open Access

Supporting Information

for *Adv. Sci.*, DOI 10.1002/advs.202104855

High-Performance and Reliable White Organic Light-Emitting Fibers for Truly Wearable Textile Displays

*Yong Ha Hwang, Byeongju Noh, Junwoo Lee, Ho Seung Lee, Yongjin Park and Kyung Cheol Choi**

ADVANCED SCIENCE
Open Access

Supporting Information

for *Adv. Sci.*, DOI: 10.1002/advs.202104855

High-Performance and Reliable White Organic Light-Emitting Fibers for Truly Wearable Textile Displays

*Yong Ha Hwang, Byeongju Noh, Junwoo Lee, Ho Seung Lee, Yongjin Park and Kyung Cheol Choi**

Supporting Information

High-Performance and Reliable White Organic Light-Emitting Fibers for Truly Wearable Textile Displays

*Yong Ha Hwang, Byeongju Noh, Junwoo Lee, Ho Seung Lee, Yongjin Park and Kyung Cheol Choi**

Y. H. Hwang, B. Noh, J. Lee, H. S. Lee, Y. Park, Prof. K. C. Choi

School of Electrical Engineering,
Korea Advanced Institute of Science and Technology,
Daejeon 34141, Republic of Korea.
*E-mail: kyungcc@kaist.ac.kr

Table section and curves

Table S1. Comparison of previously reported fiber-based light-emitting devices (fiber OLEDs); optoelectronic performance and characteristic.

Devices	Fiber type	color	Brightness $_{max}$ (cd m^{-2})	CE $_{max}$ (cd A^{-1})	Water-resistance	pressure-resistance
OLED[1]	Rectangular	Green	~ 4300 (5 V)	~46	7 h	
OLED[2]	Cylinder	Green	-	-	-	-
OLED[3]	Cylinder	Green	194.1 (> 13 V)	~ 6.84 (> 9 V)	-	-
OLED a)[4]	Cylinder	Yellow	1,459 (10 V)	2.94 (6 V)	-	-
OLED b)[5]	Cylinder	Yellow	13,937 (8.5 V)	11.6 (7.5 V)	-	-
		Red	4,462 (7 V)	16.3 (4.5 V)		-
OLED c)[6]	Cylinder	Green	11,482 (6.5 V)	60.8 (4.5 V)	-	
		Blue	1,199 (6 V)	16.9 (4 V)		
**WOLED [This work]**	**Cylinder**	**White**	**738 (6 V)**	**10.8 (4 V)**	**< 300 min**	**< 100 cycles**

a),b),c) our previous works.

Table S2. TCS values and CRI value, which were calculated by ray-tracing simulator (LightTools)

TCS (test color samples)	Value
TCS1 [7.5 R 6/4; Light Greyish Red]	96.62
TCS2 [5 Y 6/4; Dark Greyish Yellow]	88.14
TCS3 [5 GY 6/8; Strong Yellow Green]	69.82
TCS4 [2.5 G 6/6; Moderate Yellowish Green]	76.90
TCS5 [10 BG 6/4; Light Bluish Green]	92.76
TCS6 [5 PB 6/8; Light Blue]	79.68
TCS7 [2.5 P 6/8; Light Violet]	72.63
TCS8 [10 P 6/8; Light Reddish Purple]	65.15
**CRI**	**80.02**

Optical modeling of the fiber WOLED is shown in Figure S7 in detail. In the optical modeling, the measured spectrum of the fiber WOLED was applied to the light source.

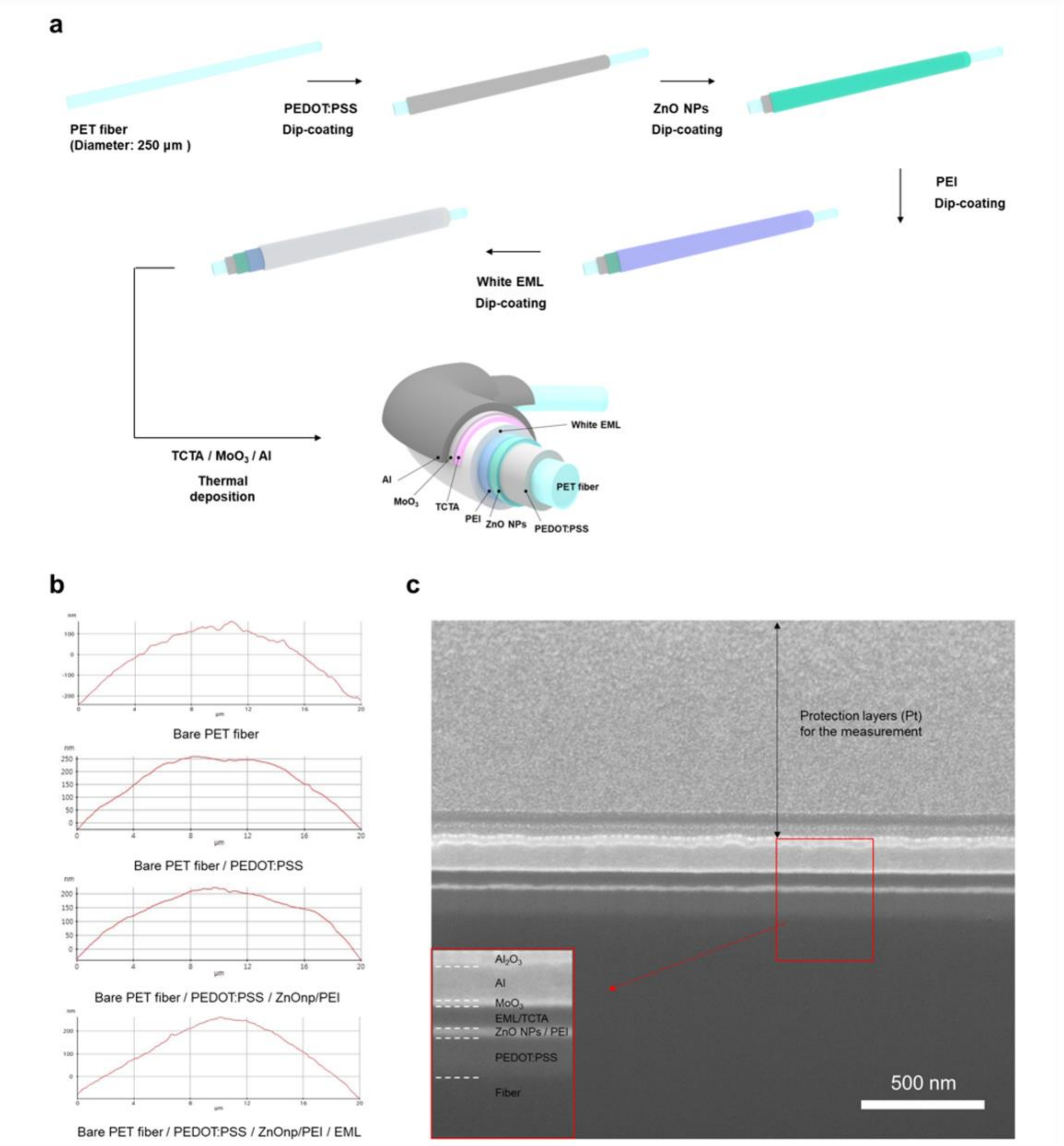

Figure S1. a) Schematic illustration of the fabrication scheme: the cathode and the electron injection layer, the emission layer, and the thermally deposited layer, which are the hole-transport layer, the hole-injection layer, and the anode. b) Line profiles of the substrate and films, as measured by an atomic force measurement system (XE-100, Park System). c) Large-area image of Figure 1e, showing the evenly deposited film.

As shown in Figure S1b, the first layer, i.e., PEDOT:PSS, facilitated planarization and acted as an electrode simultaneously. The roughness of the bare PET fiber was reduced, enabling the OLED to operate.

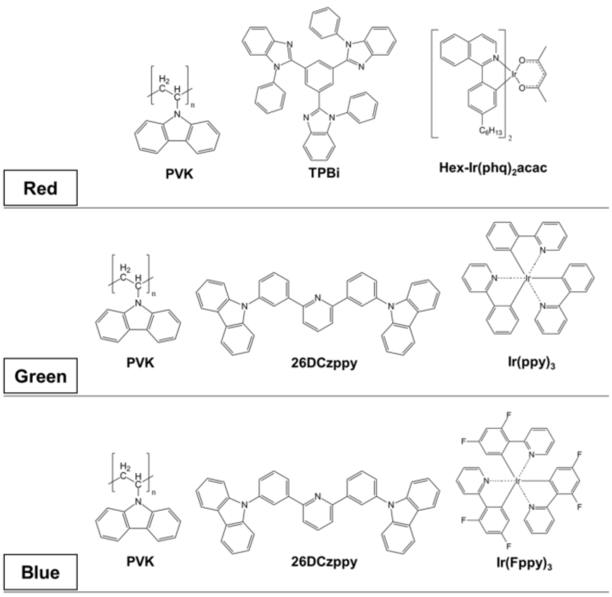

Figure S2. Materials for the white emission layer (EML) consisting of red, green, and blue components: red solution consisting of PVK, TPBi, and Hex-Ir(phq)$_2$acac; a green solution consisting of PVK, 26DCzppy, and Ir(ppy)$_3$; and a blue solution consisting of PVK, 26DCzppy, and Ir(Fppy)$_3$.[6]

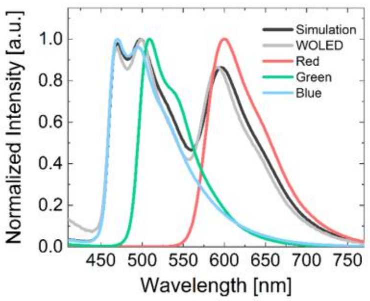

Figure S3. Simulated EL intensity of the fiber WOLED, the measured EL intensity of the fiber WOLED, and each EL intensity of RGB fiber OLED.

Under the assumption that there is no cavity effect and no formation of exciplex, the EL intensity of the WOLED can be presented as a linear combination consisting of red, green, and blue EL intensities:

$$W(\lambda)=\alpha\cdot R(\lambda)+\beta\cdot G(\lambda)+\gamma\cdot B(\lambda) \quad (1)$$

$$CIE(x) = \frac{\sum_{\lambda=380}^{780} x(\lambda)\cdot W(\lambda)}{\sum_{\lambda=380}^{780} x(\lambda)\cdot W(\lambda)+\sum_{\lambda=380}^{780} y(\lambda)\cdot W(\lambda)+\sum_{\lambda=380}^{780} z(\lambda)\cdot W(\lambda)} \quad (2)$$

$$CIE(y) = \frac{\sum_{\lambda=380}^{780} y(\lambda)\cdot W(\lambda)}{\sum_{\lambda=380}^{780} x(\lambda)\cdot W(\lambda)+\sum_{\lambda=380}^{780} y(\lambda)\cdot W(\lambda)+\sum_{\lambda=380}^{780} z(\lambda)\cdot W(\lambda)} \quad (3)$$

$x(\lambda)$, $y(\lambda)$,$and\ z(\lambda)\ are\ the\ color\ matching\ function\ at\ CIE$ 1931

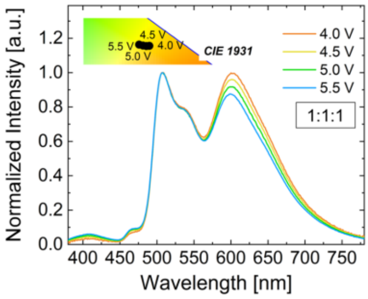

Figure S4. Electroluminescence (EL) intensity in the case of the volume ratio (1:1:1)

In the case of the volume ratio (1:1:1), the main peak (470 nm) of the blue emission was rarely measured because energy transfer (ET) is mainly performed toward the many red states with low energy from green and blue with high energy states.

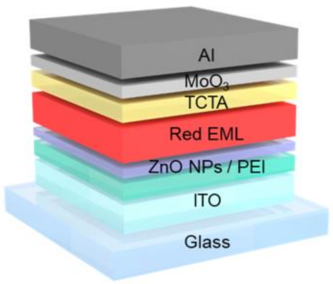

Figure S5. Illustration of glass-based red OLED indicating the detail configuration. [6]

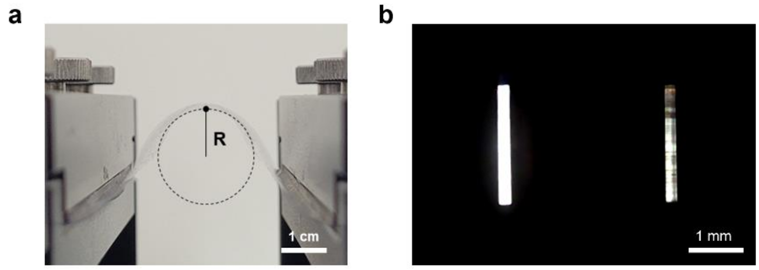

Figure S6. a) Bending machine and bending radius (R), and b) a microscopic image of the fiber WOLEDs before and after a cyclic bending test with 1000 cycles under 2 % tensile strain

Under 2% tensile strain, the fiber WOLED exhibited dark spots (stripe type) that arose in the transverse direction of the fiber, which is the direction in which the strain was applied, after a cyclic bending test with 1000 cycles. When the specimen was bent, tensile strain applied to the WOLED on the fiber can be calculated by solving t/2R, where t is the thickness of the fiber and R is the bending radius of curvature. [7] In the bending test, 1%, 1.5%, and 2% tensile strain levels were induced at radius of curvature levels of 12.5 mm, 8.3 mm, and 6.25 mm, respectively.

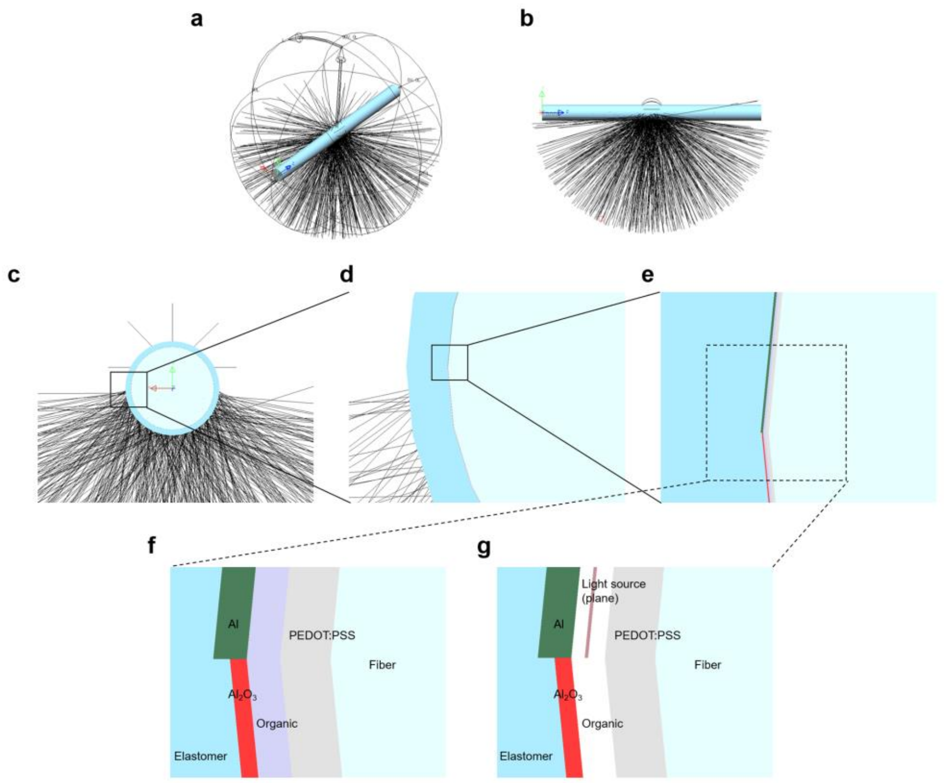

Figure S7. Ray-tracing simulation using the LightTools a) an eagle view image of the modeling b) a side view image of the modeling c) a cross-section view image of the modeling d-g) a magnified cross-section view image

Optical modeling of the fiber WOLED consisted of the fiber, PEDOT:PSS, an organic material, Al (a reflector), Al_2O_3, an elastomer object, and a plane-light source. All of the objects had a cylindrical shape with realistic thickness. The light source was immersed in the organic object. The constant refractive index of all of the objects was from previous literature and experimental results (n_{PET}: 1.57, n_{glass}: 1.52, $n_{organic(PVK)}$:1.68, n_{Al2O3}: 1.64, $n_{ecoflex}$: 1.4). In the case that 2 million rays were incident, the number of outcoupling rays was almost the same in both cases.

It was confirmed that there is the difference induced by the refractive index (n_{PET}: 1.57, n_{glass}: 1.52), resulting in a reduction of the maximum radiant intensity of about 6% when the PET was used.

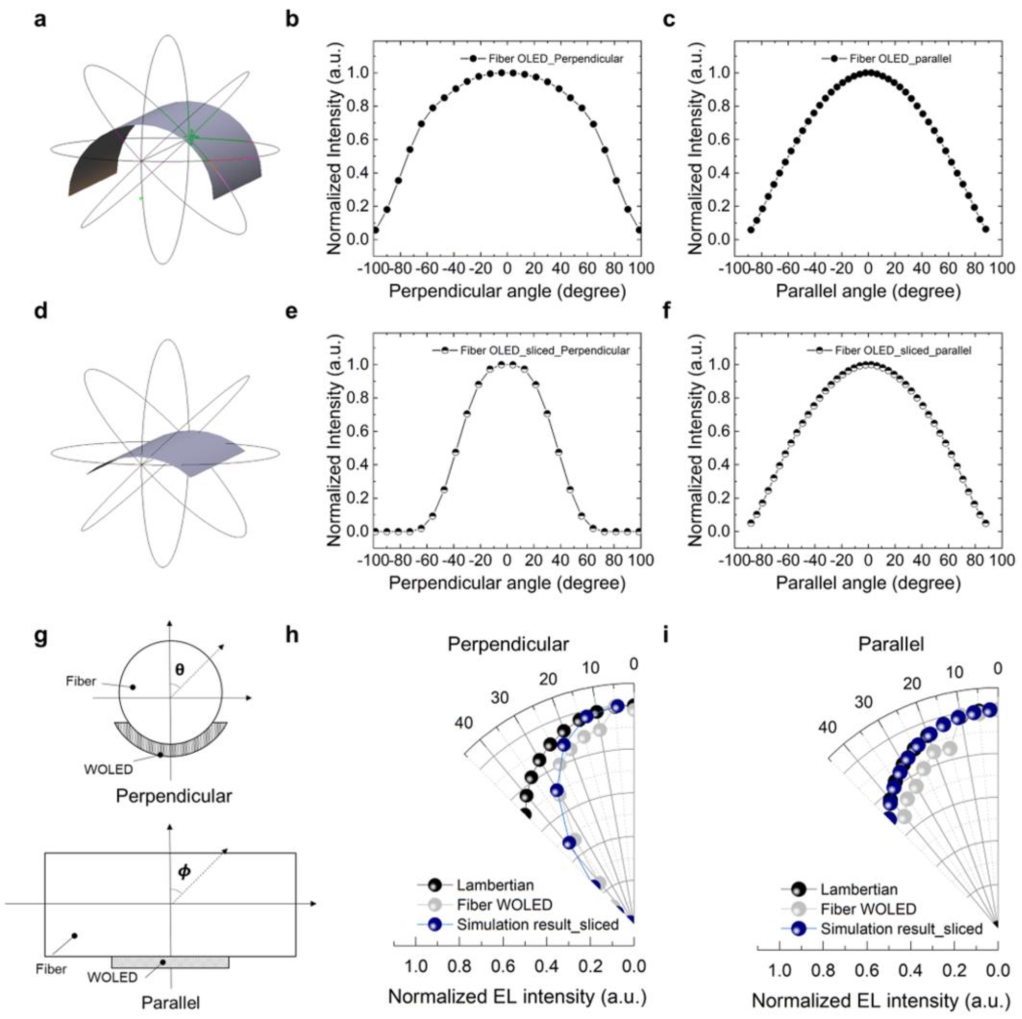

Figure S8. Comparison between the measured angular distribution and the simulated angular distribution: a) cylindrical light source, b) angular distribution using the cylindrical light source in the perpendicular direction, c) angular distribution using the cylindrical light source in the parallel direction, d) sliced cylindrical light source, e) angular distribution using the sliced cylindrical light source in the perpendicular direction, and f) angular distribution using the sliced cylindrical light source in the parallel direction g) Illustration of the fiber WOLED with a sliced light source in each direction, h) measured and simulated EL intensity levels in the perpendicular (θ) direction, and i) measured and simulated EL intensity in the parallel (ϕ) direction

There is enhanced light emittance that is induced by the circular shape of the fiber. As shown in Figure S8b, unlike the parallel direction showing Lambertian emission, light emission in the perpendicular direction was reinforced. With this simulation

result, calculations found that the outcoupling efficiency of the fiber OLED, according to our simulation, increased by nearly 2.1% compared to that of the planar OLED. However, although the angular distribution in the parallel direction is identical, there is still a difference in the perpendicular direction between the measured angular distribution and the simulated angular distribution. With the enhanced light emission from the circular shape, the effect of the charge imbalance of the side caused by the different thin layer thickness of the side was apparent. To verify this in our simulation, it was simulated that the light-emission in the case of that there was no emission in the two sides of the fiber (about half), based on the assumption that there was rarely emission because of the charge imbalance of the thin side. As a result, as shown in Figure S8e, the angular distribution of the fiber OLED using the sliced light source was similar to the measured angular distribution. In the vicinity of 0 degrees, it was observed that the maximum radiant intensity decreased by approximately 13%.

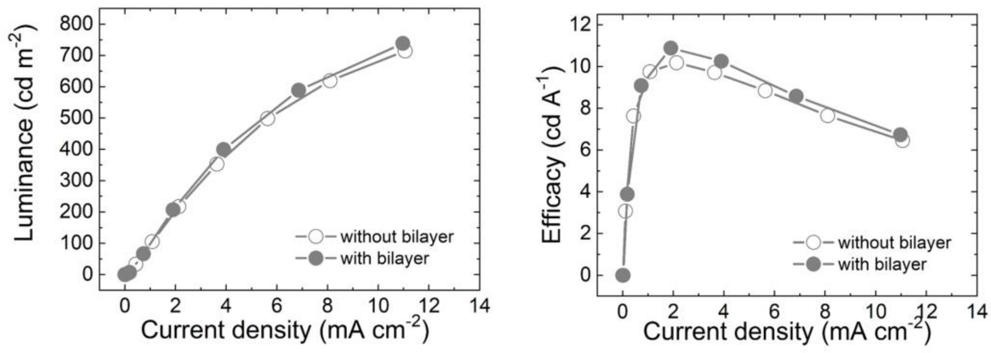

Figure S9. Comparison of the optical performance without or with the bilayer

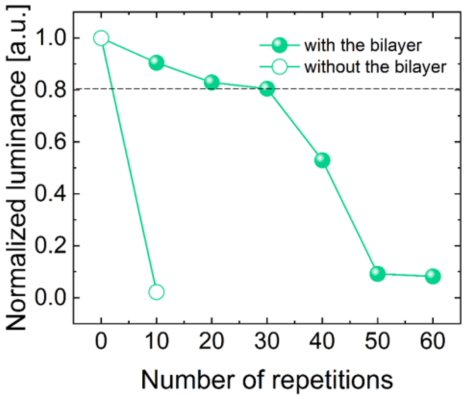

Figure S10. Repetitive load conditions using ≈5 N

In a harsh condition (≈5 N) rather than the load condition of ≈1 N, the fiber WOLED can be operated under 30 cyclic loads without dark spots.

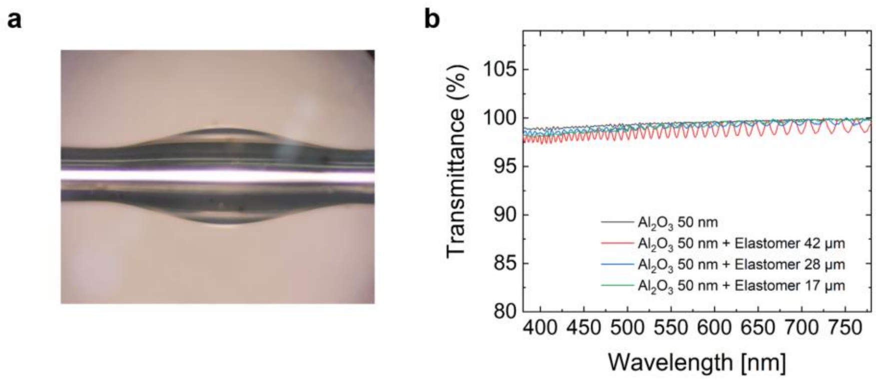

Figure S11. a) Bead-shaped elastomer b) Transmittance according to elastomer thickness

In the case of a fast withdrawal velocity, the elastomer film was thicker than in the case of a slow withdrawal velocity. If the coating is thicker than a certain level, a bead-shaped elastomer is produced due to a pulling phenomenon before curing. Furthermore, it was confirmed that a sinusoidal wave according to the wavelength was generated when the thickness of the elastomer film increased. These results mean that a relatively thick elastomer layer can induce uneven light extraction.

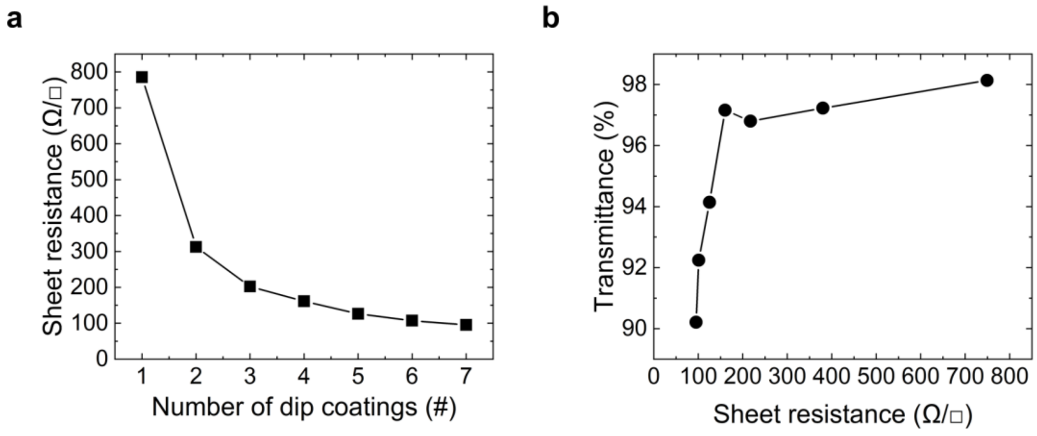

Figure S12. a) Sheet resistance of the electrode depending on number of dip coatings (the transmittance baseline: glass), and b) transmittance versus the sheet resistance curves of the electrode

Normally, there is a trade-off relationship between the transmittance and the sheet resistance of a transparent electrode due to the thickness factor. As shown in the two curves, as the thickness increases, the sheet resistance gradually decreases and saturation occurs.

Conversely, the saturated transmittance, which occurs at a thin thickness, decreases sharply with an increase in the thickness. Therefore, in the relationship, the optimized point. i.e., 107.5 Ω sq^{-1} sheet resistance and 92.2% transmittance (T), was selected (T_{ITO}: 96.5%, baseline: glass).

A UV-vis spectrophotometer (UV-2550, Shimadzu Inc.) was used to measure the transmittance of the PEDOT:PSS electrode. The sheet resistance was calculated using the equation $R_s = R \cdot t \cdot L \cdot A^{-1}$, where R was measured using a two-probe measurement device, t is the thickness of the electrode, L is the length of the electrode, and A is the cross-sectional area of the electrode.

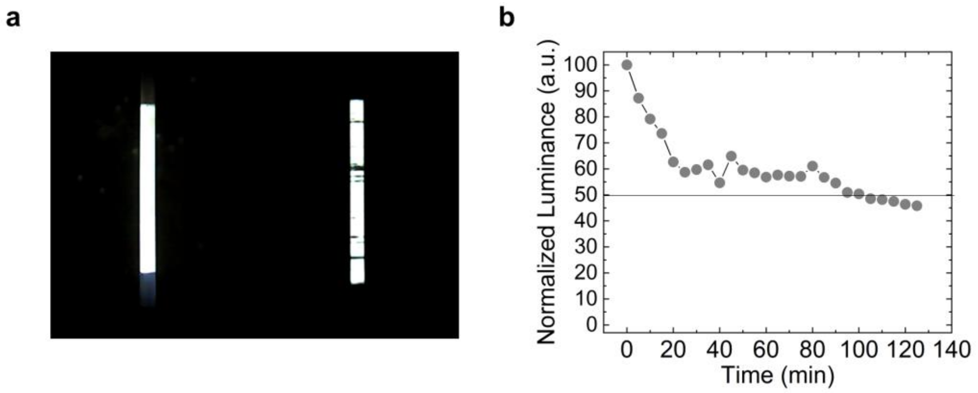

Figure S13. a) Microscope image of the fiber WOLED with (left) and without (right) encapsulation after exposure to air for ten minutes, and b) the operatin g lifetime of the encapsulated fiber WOLED (initial luminance: 50 nits)

Supporting information references

[1] Y. J. Song, J. W. Kim, H. E. Cho, Y. H. Son, M. H. Lee, J. Lee, K. C. Choi, S. M. Lee, *ACS Nano* **2020**, *14*, 1133.

[2] B. O'Connor, K. H. An, Y. Zhao, K. P. Pipe, M. Shtein, *Adv. Mater.* **2007**, *19*, 3897.

[3] B. Ki, D. Hun, T. Whan, *Nano Energy* **2020**, *70*, 104503.

[4] S. Kwon, W. Kim, H. Kim, S. Choi, B.-C. Park, S.-H. Kang, K. C. Choi, *Adv. Electron. Mater.* **2015**, *1*, 1500103.

[5] S. Kwon, H. Kim, S. Choi, E. G. Jeong, D. Kim, S. Lee, H. S. Lee, Y. C. Seo, K. C. Choi, *Nano Lett.* **2018**, *18*, 347.

[6] Y. H. Hwang, S. Kwon, J. Bin Shin, H. Kim, Y. H. Son, H. S. Lee, B. Noh, M. Nam, K. C. Choi, *Adv. Funct. Mater.* **2021**, *2009336*, 1.

[7] M. S. White, M. Kaltenbrunner, E. D. Głowacki, K. Gutnichenko, G. Kettlgruber, I. Graz, S. Aazou, C. Ulbricht, D. A. M. Egbe, M. C. Miron, Z. Major, M. C. Scharber, T. Sekitani, T. Someya, S. Bauer, N. S. Sariciftci, *Nat. Photonics* **2013**, *7*, 811.
